# Supplementary material for: Applicability and perspectives for DNA barcoding of soil invertebrates
Source: PeerJ. 2024 Jul 24;12:e17709. doi: 10.7717/peerj.17709 (PMC13043172; doi:10.7717/peerj.17709)
Supplement: Supplemental Information 1 [file peerj-12-17709-s001.pdf]

Supplemental Table S1. NCBI accession numbers and BOLD records of (A) 28S and (B) COI sequences used in this study.

(A)

genus	species	NCBI accession numbers		
Ceratophysella	denticulata	KF684371	to	KF684424
Folsomia	quadrioculata	KF684425	to	KF684480
Oppiella	nova	KF293514	to	KF293639

(B)

Collembola

genus	species	NCBI accession numbers			BOLD record		
Ceratophysella	bengtssoni	KT604455					
Ceratophysella	bengtssoni	KT604511					
Ceratophysella	bengtssoni	KT604772					
Ceratophysella	bengtssoni	KT605154					
Ceratophysella	bengtssoni	KT606390					
Ceratophysella	bengtssoni	KT606675					
Ceratophysella	bengtssoni	KT607623					
Ceratophysella	bengtssoni	MG311538					
Ceratophysella	bengtssoni	MG312803					
Ceratophysella	bengtssoni	MG313923					
Ceratophysella	bengtssoni	MG314680					
Ceratophysella	bengtssoni	MG316132					
Ceratophysella	bengtssoni	MG316593					
Ceratophysella	bengtssoni	MG318406					
Ceratophysella	bengtssoni	MG318574					
Ceratophysella	bengtssoni	MG319029					
Ceratophysella	bengtssoni	MG320085					
Ceratophysella	bengtssoni				NORCO464	to	NORCO466
Ceratophysella	communis	MN432696	to	MN432705			
Ceratophysella	communis	MN432709	to	MN432711			
Ceratophysella	communis	MN432718	to	MN432723			
Ceratophysella	communis	MN432826	to	MN432850			
Ceratophysella	comosa	AB740020	to	AB740026			
Ceratophysella	denticulata				DKINV007	to	DKINV010
Ceratophysella	denticulata	KF684537	to	KF684589			
Ceratophysella	denticulata				NORCO056	to	NORCO060
Ceratophysella	denticulata				TBGMI001	to	
Ceratophysella	granulata	HM399007	to	HM399010			
Ceratophysella	granulata	JX261875					
Ceratophysella	granulata				NORCO405		
Ceratophysella	granulata				TBGMI023		
Ceratophysella	liguladorsi	LC406886	to	LC406891			
Ceratophysella	liguladorsi	MN432730	to	MN432733			
Ceratophysella	liguladorsi	MN432772	to	MN432779			
Ceratophysella	longispina				ABSSI1724	to	ABSSI1725
Ceratophysella	longispina				ABSSI703		
Ceratophysella	longispina				ACHAR3791		
Ceratophysella	longispina				ACHAR3832		

Ceratophysella	longispina				ACHAR3834		
Ceratophysella	longispina				ACHAR3842		
Ceratophysella	longispina				ACHAR434		
Ceratophysella	longispina				DNARA1768		
Ceratophysella	longispina				DNARA383		
Ceratophysella	longispina				FCHAR1012		
Ceratophysella	longispina				FCHAR4044		
Ceratophysella	longispina				FCHAR4047		
Ceratophysella	longispina				FCHAR4048		
Ceratophysella	longispina				FCHAR4058		
Ceratophysella	longispina				FCHAR4150		
Ceratophysella	longispina				FCHAR4151		
Ceratophysella	longispina				FCHAR442		
Ceratophysella	longispina				FCHAR4959		
Ceratophysella	longispina				FCHAR4970		
Ceratophysella	longispina				GCHAR029		
Ceratophysella	longispina				GCHAR030		
Ceratophysella	longispina				GCHAR136		
Ceratophysella	longispina				GCHAR138		
Ceratophysella	longispina				GCHAR139		
Ceratophysella	longispina				GCHAR190		
Ceratophysella	longispina	MN666346					
Ceratophysella	longispina	MN667550					
Ceratophysella	longispina	MN667879					
Ceratophysella	longispina	MN668057					
Ceratophysella	longispina	MN668570					
Ceratophysella	longispina	MN669593					
Ceratophysella	longispina	MN669613					
Ceratophysella	longispina	MN670239					
Ceratophysella	longispina	MN670744					
Ceratophysella	longispina	MN671760					
Ceratophysella	longispina	MN673155					
Ceratophysella	longispina	MN674596					
Ceratophysella	longispina	MN675781					
Ceratophysella	longispina	MN675922					
Ceratophysella	longispina	MN676995					
Ceratophysella	longispina	MN677614					
Ceratophysella	longispina	MN677999					
Ceratophysella	longispina	MN678426					
Ceratophysella	longispina	MN678473					
Ceratophysella	longispina	MN678489					
Ceratophysella	longispina	MN678673					
Ceratophysella	longispina	MN679638					
Ceratophysella	longispina	MN680013					
Ceratophysella	longispina	MN680609					
Ceratophysella	longispina	MN680654					
Ceratophysella	longispina	MN680758					
Ceratophysella	longispina	MN680829					

Ceratophysella	longispina	MN682142					
Ceratophysella	longispina	MN683148					
Ceratophysella	longispina				NORCO262		
Ceratophysella	longispina				NORCO263		
Ceratophysella	longispina				SVPIT038		
Ceratophysella	pseudarmata	GU656774					
Ceratophysella	pseudarmata	GU656775					
Ceratophysella	pseudarmata	HM390611	to	HM390616			
Ceratophysella	pseudarmata	JX261792					
Ceratophysella	pseudarmata	JX261809					
Ceratophysella	pseudarmata	JX261814					
Ceratophysella	pseudarmata	JX261819					
Ceratophysella	pseudarmata	JX261843					
Ceratophysella	pseudarmata	KF641944					
Ceratophysella	pseudarmata	KF641947					
Ceratophysella	pseudarmata	KF641984					
Ceratophysella	pseudarmata	KF642024					
Ceratophysella	pseudarmata	KF642052					
Ceratophysella	pseudarmata	KF642095					
Ceratophysella	pseudarmata	KF642097					
Ceratophysella	pseudarmata	KF642106					
Ceratophysella	pseudarmata	KF642194					
Ceratophysella	pseudarmata	KF642214					
Ceratophysella	pseudarmata	KF642242					
Ceratophysella	pseudarmata	KF642283	to	KF642285			
Ceratophysella	pseudarmata	KF642305					
Ceratophysella	pseudarmata	KF642316					
Ceratophysella	pseudarmata	KF642329					
Ceratophysella	pseudarmata	KF642332					
Ceratophysella	pseudarmata	KF642352					
Ceratophysella	pseudarmata	KF642393					
Ceratophysella	pseudarmata	KF642409					
Ceratophysella	pseudarmata	KF642419					
Ceratophysella	pseudarmata	KF642423					
Ceratophysella	pseudarmata	KF642446					
Ceratophysella	pseudarmata	KF642451					
Ceratophysella	pseudarmata	KF642495					
Ceratophysella	pseudarmata	KF642510					
Ceratophysella	pseudarmata	KF642524					
Ceratophysella	pseudarmata	KF642536					
Ceratophysella	pseudarmata	KF642544					
Ceratophysella	pseudarmata	KF642554					
Ceratophysella	scotia				NORCO125	to	NORCO127
Ceratophysella	skarzynskii	MN432786	to	MN432802			
Ceratophysella	succinea	KY231136					
Ceratophysella	succinea				NORCO322		
Ceratophysella	succinea				NORCO323		
Folsomia	bisetosa	AY665330					

Folsomia	bisetosa	AY665337					
Folsomia	bisetosa	AY665350					
Folsomia	bisetosa				FCHAR4088		
Folsomia	bisetosa				FCHAR4212		
Folsomia	bisetosa				FCHAR4295		
Folsomia	bisetosa	HM893792					
Folsomia	bisetosa	KU373266					
Folsomia	bisetosa	KU374022					
Folsomia	bisetosa	KU374245					
Folsomia	bisetosa	MN668311					
Folsomia	bisetosa				NORCO209	to	NORCO211
Folsomia	candida	AB845557					
Folsomia	candida				BASS050	to	BASS054
Folsomia	candida				COLLC297	to	COLLC298
Folsomia	candida				COLLC300	to	COLLC303
Folsomia	candida				ECTTO019	to	ECTTO024
Folsomia	candida				EU330425	to	EU330426
Folsomia	candida				GENHP1348		
Folsomia	candida	GQ342255	to	GQ342258			
Folsomia	candida	HQ732049					
Folsomia	candida	JN981072					
Folsomia	candida	KF592092					
Folsomia	candida	KM978353					
Folsomia	candida	KT149648	to	KT149657			
Folsomia	candida	KU198392					
Folsomia	candida	KX351334					
Folsomia	candida	LC031886	to	LC031897			
Folsomia	ciliata				NORCO235	to	NORCO237
Folsomia	fimentaria				DKINV034	to	DKINV036
Folsomia	fimentaria	HG422609	to	HG422611			
Folsomia	fimentaria	HM390607					
Folsomia	fimentaria	KF641934					
Folsomia	fimentaria	KF641966					
Folsomia	fimentaria	KF642020					
Folsomia	fimentaria	KF642021					
Folsomia	fimentaria	KF642031					
Folsomia	fimentaria	KF642045					
Folsomia	fimentaria	KF642093					
Folsomia	fimentaria	KF642168					
Folsomia	fimentaria	KF642187					
Folsomia	fimentaria	KF642250					
Folsomia	fimentaria	KF642358					
Folsomia	fimentaria	KF642439					
Folsomia	fimentaria	KF642505					
Folsomia	fimentaria	KF642506					
Folsomia	fimentaria	LK024463					
Folsomia	fimentaria				MHCLB390	to	MHCLB392
Folsomia	fimentaria				MHCLM578		

Folsomia	fimentaria				MHCLM579		
Folsomia	fimentaria				MHCLM582		
Folsomia	fimentaria				MHCLM643		
Folsomia	nivalis				COONT299		
Folsomia	nivalis				COONT307		
Folsomia	nivalis	GU657084					
Folsomia	nivalis	GU657130					
Folsomia	nivalis	GU657131					
Folsomia	nivalis	GU657151					
Folsomia	nivalis	GU657215					
Folsomia	nivalis	GU657217					
Folsomia	nivalis	GU657232					
Folsomia	nivalis	GU657234	to	GU657237			
Folsomia	nivalis	HM390595	to	HM390600			
Folsomia	nivalis	HM893807					
Folsomia	nivalis	KF641978					
Folsomia	nivalis	KF642016					
Folsomia	nivalis	KF642105					
Folsomia	nivalis	KF642109					
Folsomia	nivalis	KF642115					
Folsomia	nivalis	KF642251					
Folsomia	nivalis	KF642326					
Folsomia	nivalis	KF642369					
Folsomia	nivalis	KF642435					
Folsomia	nivalis	KF642463					
Folsomia	nivalis	KF642474					
Folsomia	nivalis	KF642498					
Folsomia	nivalis	KF642539					
Folsomia	nivalis	KF642548					
Folsomia	nivalis	KM618202					
Folsomia	nivalis	KM619560					
Folsomia	nivalis	KM621719					
Folsomia	nivalis	MF605073					
Folsomia	nivalis	MG031345					
Folsomia	nivalis	MG031605					
Folsomia	nivalis	MG041255					
Folsomia	octoculata	HM366595	to	HM366599			
Folsomia	octoculata	LC213066	to	LC213067			
Folsomia	penicula	KT149604	to	KT149617			
Folsomia	quadrioculata				GENHP1024	to	GENHP1028
Folsomia	quadrioculata				GENHP1352		
Folsomia	quadrioculata				GENHP897	to	GENHP899
Folsomia	quadrioculata				GENHP949		
Folsomia	quadrioculata				GENHP950		
Folsomia	quadrioculata				GENHP976	to	GENHP980
Folsomia	quadrioculata	KF684590	to	KF684645			
Folsomia	quadrioculata	KJ186294	to	KJ186330			
Folsomia	quadrioculata	KJ186336	to	KJ186380			

Folsomia	quadrioculata	KT808344					
Folsomia	quadrioculata				NORCO091	to	NORCO095
Folsomia	quadrioculata				NORCO194		
Folsomia	quadrioculata				NORCO195		
Folsomia	sexoculata	AY665309					
Folsomia	sexoculata	AY665317					
Folsomia	sexoculata	AY665344					
Folsomia	sexoculata	KF642046					
Folsomia	sexoculata	KF642183					
Folsomia	sexoculata	KF642398					
Folsomia	sexoculata	KT808382					
Folsomia	sexoculata	KU373448					
Folsomia	sexoculata	KU373645					
Folsomia	sexoculata	KU373684					
Folsomia	sexoculata	KU374088					
Folsomia	sexoculata	KU374201					
Folsomia	sexoculata				NORCO021	to	NORCO025
Folsomia	sexoculata				NORCO276	to	NORCO281

Oribatida

**genus**	**species**	**NCBI accession numbers**			**BOLD record**		
Achipteria	catskillensis	MF918023					
Achipteria	catskillensis	MG407853					
Achipteria	catskillensis	MG409271					
Achipteria	catskillensis	MG409812					
Achipteria	catskillensis	MG410982					
Achipteria	catskillensis	MG412805					
Achipteria	catskillensis	MG412838					
Achipteria	catskillensis	MG413783					
Achipteria	catskillensis	MG414699					
Achipteria	catskillensis	MN358863					
Achipteria	catskillensis	MN363187					
Achipteria	coleoptrata	JF263594	to	JF263734			
Achipteria	howardi	MN352130					
Achipteria	howardi	MN352148					
Achipteria	howardi	MN353448					
Achipteria	howardi	MN355391					
Aeroppia	sp	MN348335					
Aeroppia	sp	MN348663					
Aeroppia	sp	MN349125					
Aeroppia	sp	MN349482					
Aeroppia	sp	MN351728					
Aeroppia	sp	MN353936					
Berniella	hauseri	KF293512					
Berniella	hauseri	KF293513					
Dissorhina	ornata				GRAFW2940		
Dissorhina	ornata				TBGMI159		
Dissorhina	ornata				UZINS164	to	UZINS169

Dissorhina	ornata	JX833940					
Dissorhina	ornata	MZ623773					
Dissorhina	ornata	MZ624392					
Dissorhina	ornata	MZ624781					
Dissorhina	ornata	MZ626334					
Dissorhina	ornata	MZ628959					
Multioppia	sp	MF917371					
Multioppia	sp	MG408409					
Multioppia	sp	MN348018					
Multioppia	sp	MN350500					
Multioppia	sp	MN352376					
Multioppia	sp	MN355276					
Nothrus	anauniensis	KP979344					
Nothrus	anauniensis	MN348214					
Nothrus	anauniensis	MN349924					
Nothrus	anauniensis	MN350333					
Nothrus	anauniensis	MN352954					
Nothrus	anauniensis	MN356569					
Nothrus	anauniensis	MN356874					
Nothrus	anauniensis	MN358235					
Nothrus	anauniensis	MN358359					
Nothrus	anauniensis	MN358825					
Nothrus	anauniensis	MN359209					
Nothrus	anauniensis	MN360978					
Nothrus	anauniensis	MN361141					
Nothrus	anauniensis	MN362171					
Nothrus	anauniensis	MN362314					
Nothrus	anauniensis	MN362431					
Nothrus	anauniensis	MN362542					
Nothrus	anauniensis	MN362623					
Nothrus	anauniensis	MN363763					
Nothrus	anauniensis	MN364153					
Nothrus	borussicus	KM828933					
Nothrus	borussicus	KM832928					
Nothrus	borussicus	KM839522					
Nothrus	borussicus	MN355393					
Nothrus	borussicus	MN355457					
Nothrus	borussicus	MN355471					
Nothrus	borussicus	MZ626988					
Nothrus	borussicus	OK065582					
Nothrus	palustris	JF263837	to	JF263840			
Nothrus	palustris	MZ609240					
Nothrus	palustris	MZ609306					
Nothrus	palustris	MZ609791					
Nothrus	palustris	MZ609929					
Nothrus	palustris	MZ610164					
Nothrus	palustris	MZ610775					
Nothrus	pratensis	HQ941502	to	HQ941504			

Nothrus	pratensis	JX835667					
Nothrus	pratensis	JX835760					
Nothrus	silvestris	JF263737	to	JF263836			
Oppia	nitens	KT702890					
Oppia	nitens	KT704526					
Oppia	nitens	KT705588					
Oppia	nitens	MF909508					
Oppia	nitens	MF909548					
Oppia	nitens	MF909833					
Oppia	nitens	MF909870					
Oppia	nitens	MF910574					
Oppia	nitens	MF910962					
Oppia	nitens	MF912317					
Oppia	nitens	MF912481					
Oppia	nitens	MF914885					
Oppia	nitens	MF915887					
Oppia	nitens	MF916397					
Oppia	nitens	MF916423					
Oppia	nitens	MG407958					
Oppia	nitens	MG408067					
Oppia	nitens	MG408199					
Oppia	nitens	MG408516					
Oppia	nitens	MG409011					
Oppia	nitens	MG409387					
Oppia	nitens	MG409555					
Oppia	nitens	MG409741					
Oppia	nitens	MG409957					
Oppia	nitens	MG410228					
Oppia	nitens	MG410231					
Oppia	nitens	MG410450					
Oppia	nitens	MG410542					
Oppia	nitens	MG410641					
Oppia	nitens	MG410665					
Oppia	nitens	MG410756					
Oppia	nitens	MG410933					
Oppia	nitens	MG411030					
Oppia	nitens	MG411338					
Oppia	nitens	MG411408					
Oppia	nitens	MG411639					
Oppia	nitens	MG411679					
Oppia	nitens	MG411951					
Oppia	nitens	MG411956					
Oppia	nitens	MG411989					
Oppia	nitens	MG412148					
Oppia	nitens	MG412627					
Oppia	nitens	MG412854					
Oppia	nitens	MG413003					
Oppia	nitens	MG413017					

Oppia	nitens	MG413275		
Oppia	nitens	MG413348		
Oppia	nitens	MG413863		
Oppia	nitens	MG414211		
Oppia	nitens	MG414439		
Oppia	nitens	MG414452		
Oppia	nitens	MG414496		
Oppia	nitens	MG415603		
Oppia	nitens	MG415631		
Oppia	nitens	MG416254		
Oppia	nitens	MG416481		
Oppia	nitens	MG416595		
Oppia	nitens	MG416678		
Oppia	nitens	MG416757		
Oppia	nitens	MG416820		
Oppia	nitens	MN347410		
Oppia	nitens	MN347540		
Oppia	nitens	MN347772		
Oppia	nitens	MN347882		
Oppia	nitens	MN347896		
Oppia	nitens	MN348470		
Oppia	nitens	MN348610		
Oppia	nitens	MN349650		
Oppia	nitens	MN349776		
Oppia	nitens	MN350427		
Oppia	nitens	MN350563		
Oppia	nitens	MN350810		
Oppia	nitens	MN351606		
Oppia	nitens	MN351611		
Oppia	nitens	MN352679		
Oppia	nitens	MN352935		
Oppia	nitens	MN353946		
Oppia	nitens	MN354228		
Oppia	nitens	MN354365		
Oppia	nitens	MN354940		
Oppia	nitens	MN355019		
Oppia	nitens	MN358307		
Oppia	nitens	MN358408		
Oppia	nitens	MN359598		
Oppia	nitens	MN360119		
Oppia	nitens	MN360531		
Oppia	nitens	MN360836		
Oppia	nitens	MN361228		
Oppia	nitens	MN362461		
Oppia	nitens	MN363593		
Oppia	nitens	MSSCA950		
Oppia	sp	MN353203		
Oppia	sp	MN354195		

Oppia	sp	MN354851					
Oppiella	nova	KF293402	to	KF293511			
Oppiella	nova	KF29370	to	KF29376			
Oppiella	subpectinata	MZ625745					
Oppiella	uliginosa	MZ623733					
Oppiella	uliginosa	MZ626811					
Oppiella	uliginosa	MZ626812					
Platynothrus	peltifer	JF263841	to	JF264000			
Platynothrus	capillatus				GRAFW2963		
Platynothrus	capillatus				GRAFW2964	to	GRAFW2966
Platynothrus	thori				GRAFW2205	to	GRAFW2208
Platynothrus	yamasakii	KM824246					
Platynothrus	yamasakii	KM824521					
Platynothrus	yamasakii	KM824751					
Platynothrus	yamasakii	KM825213					
Platynothrus	yamasakii	KM825397					
Platynothrus	yamasakii	KM825820					
Platynothrus	yamasakii	KM826566					
Platynothrus	yamasakii	KM827258					
Platynothrus	yamasakii	KM827351					
Platynothrus	yamasakii	KM827614					
Platynothrus	yamasakii	KM827842					
Platynothrus	yamasakii	KM828293					
Platynothrus	yamasakii	KM828485					
Platynothrus	yamasakii	KM828488					
Platynothrus	yamasakii	KM828514					
Platynothrus	yamasakii	KM828713					
Platynothrus	yamasakii	KM829699					
Platynothrus	yamasakii	KM831977					
Platynothrus	yamasakii	KM832326					
Platynothrus	yamasakii	KM832405					
Platynothrus	yamasakii	KM832456					
Platynothrus	yamasakii	KM832742					
Platynothrus	yamasakii	KM833022					
Platynothrus	yamasakii	KM834709					
Platynothrus	yamasakii	KM835532					
Platynothrus	yamasakii	KM835709					
Platynothrus	yamasakii	KM836024					
Platynothrus	yamasakii	KM836447					
Platynothrus	yamasakii	KM837344					
Platynothrus	yamasakii	KM837927					
Platynothrus	yamasakii	KM837936					
Platynothrus	yamasakii	KM839624					
Platynothrus	yamasakii	MF908595					
Platynothrus	yamasakii	MF908985					
Platynothrus	yamasakii	MF909070					
Platynothrus	yamasakii	MF909123					
Platynothrus	yamasakii	MF909159					

Platynothrus	yamasakii	MF909258		
Platynothrus	yamasakii	MF909408		
Platynothrus	yamasakii	MF910162		
Platynothrus	yamasakii	MF910674		
Platynothrus	yamasakii	MF910899		
Platynothrus	yamasakii	MF910950		
Platynothrus	yamasakii	MF911002		
Platynothrus	yamasakii	MF911152		
Platynothrus	yamasakii	MF911437		
Platynothrus	yamasakii	MF911989		
Platynothrus	yamasakii	MF913339		
Platynothrus	yamasakii	MF913851		
Platynothrus	yamasakii	MF913909		
Platynothrus	yamasakii	MF914062		
Platynothrus	yamasakii	MF914523		
Platynothrus	yamasakii	MF914690		
Platynothrus	yamasakii	MF914840		
Platynothrus	yamasakii	MF915423		
Platynothrus	yamasakii	MF915621		
Platynothrus	yamasakii	MF916793		
Platynothrus	yamasakii	MF916970		
Platynothrus	yamasakii	MF917386		
Platynothrus	yamasakii	MF917563		
Platynothrus	yamasakii	MF917737		
Platynothrus	yamasakii	MG414970		
Platynothrus	yamasakii	MN348290		
Platynothrus	yamasakii	MN348357		
Platynothrus	yamasakii	MN348436		
Platynothrus	yamasakii	MN348638		
Platynothrus	yamasakii	MN348640		
Platynothrus	yamasakii	MN349039		
Platynothrus	yamasakii	MN349216		
Platynothrus	yamasakii	MN349336		
Platynothrus	yamasakii	MN349983		
Platynothrus	yamasakii	MN350241		
Platynothrus	yamasakii	MN350302		
Platynothrus	yamasakii	MN350408		
Platynothrus	yamasakii	MN350940		
Platynothrus	yamasakii	MN351687		
Platynothrus	yamasakii	MN353675		
Platynothrus	yamasakii	MN354141		
Platynothrus	yamasakii	MN354960		
Platynothrus	yamasakii	MSSCA4180		
Steganacarus	applicatus	GQ864374		
Steganacarus	applicatus	JX891544	to	JX891548
Steganacarus	applicatus	KP187550	to	KP187553
Steganacarus	applicatus	MZ624774		
Steganacarus	applicatus	MZ625711		

Steganacarus	applicatus	MZ625812		
Steganacarus	carinatus	KP187531	to	KP187537
Steganacarus	carinatus	KP187544		
Steganacarus	carinatus	KP187549		
Steganacarus	crassisetosus	JX891555	to	JX891560
Steganacarus	magnus	JF264001	to	JF264063
Steganacarus	magnus	JF264067	to	JF264084
Steganacarus	magnus	JF264092	to	JF264097
Steganacarus	magnus	JF264099	to	JF264165
Steganacarus	similis	JX891539	to	JX891543
Steganacarus	spinosus	GQ864370		
Steganacarus	spinosus	JX891549	to	JX891554
Steganacarus	spinosus	KP187524	to	KP187527
Steganacarus	spinosus	KP187538	to	KP187541
