# Supplementary material for: Applicability and perspectives for DNA barcoding of soil invertebrates
Source: PeerJ. 2024 Jul 24;12:e17709. doi: 10.7717/peerj.17709 (PMC13043172; doi:10.7717/peerj.17709)
Supplement: Supplemental Information 2 — Sequences of 28S and COI are from the same individual and were available for one Oribatida species (Oppiella nova) and two Collembola species (Ceratophysella denticulata, Folsomia quadrioculata). [file peerj-12-17709-s002.pdf]

Supplemental Table S2. Assignment of ASAP COI lineages to the respective 28S rDNA haplotypes. Sequences of 28S and COI are from the same individual and were available for one Oribatida species (*Oppiella nova*) and two Collembola species (*Ceratophysella denticulata, Folsomia quadrioculata)*

Oppiella nova

28S haplotype	Sequences belonging to haplotype 28S	ASAP lineage (COI)
1	Oppiella_nova_Ger_HA_F_1_KF293514_1	lineage_1
	Oppiella_nova_Ger_HA_F_2_KF293515_1	lineage_1
	Oppiella_nova_Ger_HA_F_3_KF293516_1	lineage_1
	Oppiella_nova_Ger_HA_F_4_KF293517_1	lineage_1
	Oppiella_nova_Ger_KW_MG_2_KF293546_1	lineage_2
	Oppiella_nova_Ger_KW_IFG_2_KF293549_1	lineage_3
	Oppiella_nova_Ger_KW_IFG_3_KF293550_1	lineage_2
	Oppiella_nova_Ger_KW_MG_8_KF293575_1	lineage_2
	Oppiella_nova_Ger_KW_MG_12_KF293579_1	lineage_2
	Oppiella_nova_Ger_KW_MG_14_KF293581_1	lineage_2
	Oppiella_nova_Ger_KW_MG_15_KF293582_1	lineages_6
	Oppiella_nova_Ger_KW_MG_17_KF293584_1	lineage_2
	Oppiella_nova_Ger_KW_MG_18_KF293585_1	lineage_2
	Oppiella_nova_Ger_KW_MG_20_KF293587_1	missing
	Oppiella_nova_Ger_KW_MG_21_KF293588_1	missing
	Oppiella_nova_Ger_KW_MG_22_KF293589_1	missing
	Oppiella_nova_Ger_KW_MG_23_KF293590_1	missing
	Oppiella_nova_Ger_SO_F_6_KF293596_1	lineage_1
	Oppiella_nova_Ger_SO_MG_2_KF293600_1	lineage_1
2	Oppiella_nova_Ger_HA_IFG_1_KF293518_1	lineage_2
	Oppiella_nova_Ger_HA_IFG_2_KF293519_1	lineage_2
	Oppiella_nova_Ger_HA_IFG_3_KF293520_1	lineage_2
	Oppiella_nova_Ger_HA_IFG_4_KF293521_1	lineage_2
	Oppiella_nova_Ger_HA_IFG_5_KF293522_1	lineage_2
	Oppiella_nova_Ger_HA_IFG_6_KF293523_1	lineage_2
	Oppiella_nova_Ger_HA_IFG_7_KF293524_1	lineage_2
	Oppiella_nova_Ger_HA_IFG_8_KF293525_1	lineage_2
	Oppiella_nova_Ger_HA_IFG_9_KF293526_1	lineage_2
	Oppiella_nova_Ger_HA_IFG_10_KF293527_1	missing
	Oppiella_nova_Ger_HA_IFG_11_KF293528_1	missing
	Oppiella_nova_Ger_KW_G_1_KF293530_1	lineage_5
	Oppiella_nova_Ger_KW_G_2_KF293543_1	lineage_2
	Oppiella_nova_Ger_KW_G_3_KF293544_1	lineage_7
	Oppiella_nova_Ger_KW_G_5_KF293556_1	lineage_2
	Oppiella_nova_Ger_KW_G_6_KF293557_1	lineage_7
	Oppiella_nova_Ger_KW_G_10_KF293561_1	lineage_7
	Oppiella_nova_Ger_KW_G_11_KF293562_1	lineage_2
	Oppiella_nova_Ger_KW_G_12_KF293563_1	lineage_7
	Oppiella_nova_Ger_KW_G_14_KF293565_1	lineage_5
	Oppiella_nova_Ger_KW_G_15_KF293566_1	lineage_7
	Oppiella_nova_Ger_KW_G_18_KF293569_1	lineage_7
	Oppiella_nova_Ger_KW_G_20_KF293571_1	missing

	Oppiella_nova_Ger_KW_MG_6_KF293573_1	lineage_2
	Oppiella_nova_Ger_KW_MG_7_KF293574_1	lineage_2
	Oppiella_nova_Ger_KW_MG_9_KF293576_1	lineages_6
	Oppiella_nova_Ger_KW_MG_10_KF293577_1	lineages_6
	Oppiella_nova_Ger_KW_MG_16_KF293583_1	lineage_2
	Oppiella_nova_Ger_SO_MG_5_KF293603_1	missing
	Oppiella_nova_Ger_SO_MG_6_KF293604_1	missing
	Oppiella_nova_Ger_UE_G_1_KF293610_1	lineage_2
	Oppiella_nova_Ger_UE_G_2_KF293611_1	lineage_2
	Oppiella_nova_Ger_UE_MG_1_KF293613_1	lineage_2
	Oppiella_nova_Ger_UE_MG_2_KF293614_1	lineage_2
	Oppiella_nova_Ger_UE_MG_3_KF293615_1	lineage_2
	Oppiella_nova_Ger_UE_MG_4_KF293616_1	missing
	Oppiella_nova_Ger_UE_MG_5_KF293617_1	missing
	Oppiella_nova_Ger_UE_MG_7_KF293619_1	missing
3	Oppiella_nova_Ger_KW_F_1_KF293529_1	lineage_3
4	Oppiella_nova_Ger_KW_IFG_1_KF293532_1	lineage_2
	Oppiella_nova_Ger_KW_F_6_KF293537_1	lineage_1
	Oppiella_nova_Ger_KW_F_10_KF293541_1	lineage_1
	Oppiella_nova_Ger_KW_MG_11_KF293578_1	lineage_2
	Oppiella_nova_Ger_UE_IFG_9_KF293628_1	lineage_1
5	Oppiella_nova_Ger_KW_F_2_KF293533_1	lineage_3
6	Oppiella_nova_Ger_KW_F_3_KF293534_1	lineage_3
7	Oppiella_nova_Ger_KW_F_4_KF293535_1	lineage_1
	Oppiella_nova_Ger_KW_F_14_KF293554_1	missing
	Oppiella_nova_Ger_KW_MG_13_KF293580_1	lineage_2
	Oppiella_nova_Ger_KW_MG_19_KF293586_1	lineage_2
	Oppiella_nova_Ger_TW_F_1_KF293608_1	lineage_8
	Oppiella_nova_Ger_UE_IFG_7_KF293626_1	lineage_1
8	Oppiella_nova_Ger_KW_F_5_KF293536_1	lineage_4
9	Oppiella_nova_Ger_KW_F_7_KF293538_1	lineage_3
10	Oppiella_nova_Ger_KW_F_8_KF293539_1	lineage_4
11	Oppiella_nova_Ger_KW_F_9_KF293540_1	lineage_4
	Oppiella_nova_Ger_KW_F_13_KF293553_1	missing
	Oppiella_nova_Ger_TW_F_2_KF293606_1	lineage_3
	Oppiella_nova_Ger_UE_IFG_3_KF293622_1	lineage_1
	Oppiella_nova_Ger_UE_IFG_13_KF293632_1	lineage_3
12	Oppiella_nova_Ger_KW_MG_1_KF293545_1	lineage_2
	Oppiella_nova_Ger_KW_MG_3_KF293547_1	lineage_6
	Oppiella_nova_Ger_KW_MG_4_KF293548_1	lineage_2
13	Oppiella_nova_Ger_KW_F_11_KF293551_1	lineage_3
14	Oppiella_nova_Ger_KW_F_12_KF293552_1	lineage_1
15	Oppiella_nova_Ger_KW_G_4_KF293555_1	lineage_2
	Oppiella_nova_Ger_KW_G_8_KF293559_1	lineage_7
	Oppiella_nova_Ger_KW_G_9_KF293560_1	lineage_7
	Oppiella_nova_Ger_KW_G_13_KF293564_1	lineage_2
	Oppiella_nova_Ger_KW_G_16_KF293567_1	lineage_7
	Oppiella_nova_Ger_KW_G_17_KF293568_1	lineage_2

16	Oppiella_nova_Ger_KW_G_7_KF293558_1	lineage_7
	Oppiella_nova_Ger_KW_MG_5_KF293572_1	lineage_6
	Oppiella_nova_Ger_SO_F_8_KF293598_1	missing
	Oppiella_nova_Ger_UE_G_3_KF293612_1	lineage_6
	Oppiella_nova_Ger_UE_MG_6_KF293618_1	missing
17	Oppiella_nova_Ger_KW_G_19_KF293570_1	lineage_7
18	Oppiella_nova_Ger_SO_F_1_KF293591_1	lineage_3
19	Oppiella_nova_Ger_SO_F_2_KF293592_1	lineage_1
	Oppiella_nova_Ger_SO_F_3_KF293593_1	lineage_3
	Oppiella_nova_Ger_SO_F_4_KF293594_1	lineage_1
	Oppiella_nova_Ger_UE_IFG_19_KF293638_1	lineage_1
20	Oppiella_nova_Ger_SO_F_5_KF293595_1	lineage_1
21	Oppiella_nova_Ger_SO_F_7_KF293597_1	lineage_1
	Oppiella_nova_Ger_UE_IFG_4_KF293623_1	lineage_1
	Oppiella_nova_Ger_UE_IFG_15_KF293634_1	lineage_1
22	Oppiella_nova_Ger_SO_MG_1_KF293599_1	lineage_2
	Oppiella_nova_Ger_SO_MG_4_KF293602_1	lineage_2
23	Oppiella_nova_Ger_SO_MG_3_KF293601_1	lineage_1
24	Oppiella_nova_Ger_TW_F_1_KF293605_1	lineage_8
25	Oppiella_nova_Ger_TW_F_3_KF293607_1	lineage_8
26	Oppiella_nova_Ger_TW_IFG_1_KF293609_1	missing
27	Oppiella_nova_Ger_UE_IFG_1_KF293620_1	lineage_3
28	Oppiella_nova_Ger_UE_IFG_2_KF293621_1	lineage_1
	Oppiella_nova_Ger_UE_IFG_11_KF293630_1	lineage_1
29	Oppiella_nova_Ger_UE_IFG_5_KF293624_1	lineage_1
	Oppiella_nova_Ger_UE_IFG_6_KF293625_1	lineage_1
	Oppiella_nova_Ger_UE_IFG_8_KF293627_1	lineage_3
	Oppiella_nova_Ger_UE_IFG_10_KF293629_1	lineage_3
	Oppiella_nova_Ger_UE_IFG_12_KF293631_1	lineage_1
	Oppiella_nova_Ger_UE_IFG_14_KF293633_1	lineage_1
	Oppiella_nova_Ger_UE_IFG_16_KF293635_1	lineage_3
	Oppiella_nova_Ger_UE_IFG_17_KF293636_1	lineage_1
	Oppiella_nova_Ger_UE_IFG_20_KF293639_1	lineage_1
30	Oppiella_nova_Ger_UE_IFG_18_KF293637_1	lineage_1

Ceratophysella denticulata

28S haplotype	Sequences belonging to haplotype 28S	ASAP lineage (COI)
1	Ceratophysella_denticulata_Aus_1_KF684375	lineage_19
	Ceratophysella_denticulata_Aus_2_KF684374	lineage_19
	Ceratophysella_denticulata_Aus_3_KF684373	lineage_19
	Ceratophysella_denticulata_Aus_4_KF684372	lineage_19
	Ceratophysella_denticulata_Aus_5_KF684371	missing
	Ceratophysella_denticulata_Fra_1_KF684383	lineage_19
	Ceratophysella_denticulata_Fra_2_KF684382	lineage_19
	Ceratophysella_denticulata_Fra_3_KF684381	lineage_19
	Ceratophysella_denticulata_Fra_4_KF684380	lineage_19
	Ceratophysella_denticulata_Fra_5_KF684379	lineage_19

2	Ceratophysella_denticulata_Cro_1_KF684378	missing
	Ceratophysella_denticulata_Cro_2_KF684377	missing
	Ceratophysella_denticulata_Cro_3_KF684376	missing
	Ceratophysella_denticulata_Ger_1_KF684388	lineage_18
	Ceratophysella_denticulata_Ger_2_KF684387	lineage_18
	Ceratophysella_denticulata_Ger_3_KF684386	lineage_18
	Ceratophysella_denticulata_Ger_4_KF684385	lineage_18
	Ceratophysella_denticulata_Ger_5_KF684384	lineage_18
	Ceratophysella_denticulata_Nor_1_KF684406	lineage_18
	Ceratophysella_denticulata_Nor_2_KF684405	lineage_18
	Ceratophysella_denticulata_Nor_3_KF684404	lineage_18
	Ceratophysella_denticulata_Nor_4_KF684403	lineage_18
	Ceratophysella_denticulata_Nor_5_KF684402	lineage_18
	Ceratophysella_denticulata_Pol_1_KF684410	lineage_18
	Ceratophysella_denticulata_Pol_2_KF684409	lineage_18
	Ceratophysella_denticulata_Pol_3_KF684408	lineage_18
	Ceratophysella_denticulata_Pol_4_KF684407	lineage_18
3	Ceratophysella_denticulata_Gre_1_KF684393	liineage_13
	Ceratophysella_denticulata_Gre_2_KF684392	liineage_13
	Ceratophysella_denticulata_Gre_3_KF684391	liineage_13
	Ceratophysella_denticulata_Gre_4_KF684390	liineage_13
	Ceratophysella_denticulata_Gre_5_KF684389	liineage_13
4	Ceratophysella_denticulata_Ita_1_KF684396	lineage_20
	Ceratophysella_denticulata_Ita_2_KF684395	lineage_20
	Ceratophysella_denticulata_Ita_3_KF684394	lineage_20
5	Ceratophysella_denticulata_Mon_1_KF684401	lineage_17
	Ceratophysella_denticulata_Mon_2_KF684400	lineage_17
	Ceratophysella_denticulata_Mon_3_KF684399	lineage_17
	Ceratophysella_denticulata_Mon_4_KF684398	lineage_17
	Ceratophysella_denticulata_Mon_5_KF684397	lineage_17
6	Ceratophysella_denticulata_Rus_1_KF684415	lineage_21
	Ceratophysella_denticulata_Rus_2_KF684414	lineage_21
	Ceratophysella_denticulata_Rus_3_KF684413	lineage_21
	Ceratophysella_denticulata_Rus_4_KF684412	lineage_21
	Ceratophysella_denticulata_Rus_5_KF684411	lineage_21
	Ceratophysella_denticulata_Ser_1_KF684420	lineage_21
	Ceratophysella_denticulata_Ser_2_KF684419	lineage_21
	Ceratophysella_denticulata_Ser_3_KF684418	lineage_21
	Ceratophysella_denticulata_Ser_4_KF684417	lineage_21
	Ceratophysella_denticulata_Ser_5_KF684416	lineage_21
7	Ceratophysella_denticulata_Spa_1_KF684424	lineage_9
	Ceratophysella_denticulata_Spa_3_KF684422	lineage_9
	Ceratophysella_denticulata_Spa_4_KF684421	lineage_9
8	Ceratophysella_denticulata_Spa_2_KF684423	lineage_9

Folsomia quadrioculata

28S haplotype	Sequences belonging to haplotype 28S	ASAP lineage (COI)

1	Folsomia_quadrioculata_Gre_1_KF684447_2	lineage_33
	Folsomia_quadrioculata_Gre_2_KF684448_2	lineage_33
	Folsomia_quadrioculata_Gre_3_KF684449_2	lineage_34
	Folsomia_quadrioculata_Gre_4_KF684450_2	lineage_34
	Folsomia_quadrioculata_Gre_5_KF684451_2	lineage_34
	Folsomia_quadrioculata_Mon_1_KF684457_2	lineage_37
	Folsomia_quadrioculata_Mon_2_KF684458_2	lineage_37
	Folsomia_quadrioculata_Mon_3_KF684459_2	lineage_37
	Folsomia_quadrioculata_Mon_4_KF684460_2	lineage_37
	Folsomia_quadrioculata_Mon_5_KF684461_2	lineage_37
2	Folsomia_quadrioculata_Nor_1_KF684462_4	lineage_29
	Folsomia_quadrioculata_Nor_2_KF684463_4	lineage_29
	Folsomia_quadrioculata_Nor_3_KF684464_4	lineage_29
	Folsomia_quadrioculata_Nor_4_KF684465_4	lineage_29
	Folsomia_quadrioculata_Nor_5_KF684466_4	lineage_29
3	Folsomia_quadrioculata_Aus_1_KF684425_3	lineage_18
	Folsomia_quadrioculata_Aus_2_KF684426_3	lineage_18
	Folsomia_quadrioculata_Aus_3_KF684427_3	lineage_18
	Folsomia_quadrioculata_Cro_1_KF684428_3	lineage_19
	Folsomia_quadrioculata_Cro_2_KF684429_3	lineage_19
	Folsomia_quadrioculata_Cro_3_KF684430_3	lineage_19
	Folsomia_quadrioculata_Cro_4_KF684431_3	lineage_20
	Folsomia_quadrioculata_Est_1_KF684432_3	lineage_23
	Folsomia_quadrioculata_Est_2_KF684433_3	lineage_23
	Folsomia_quadrioculata_Est_3_KF684434_3	lineage_23
	Folsomia_quadrioculata_Est_4_KF684435_3	lineage_23
	Folsomia_quadrioculata_Est_5_KF684436_3	lineage_23
	Folsomia_quadrioculata_Fra_1_KF684437_3	lineage_25
	Folsomia_quadrioculata_Fra_2_KF684438_3	lineage_25
	Folsomia_quadrioculata_Fra_3_KF684439_3	lineage_25
	Folsomia_quadrioculata_Fra_4_KF684440_3	lineage_25
	Folsomia_quadrioculata_Fra_5_KF684441_3	lineage_25
	Folsomia_quadrioculata_Ger_1_KF684442_3	lineage_18
	Folsomia_quadrioculata_Ger_2_KF684443_3	lineage_18
	Folsomia_quadrioculata_Ger_3_KF684444_3	lineage_18
	Folsomia_quadrioculata_Ger_4_KF684445_3	lineage_18
	Folsomia_quadrioculata_Ger_5_KF684446_3	lineage_18
	Folsomia_quadrioculata_Ita_1_KF684452_3	lineage_36
	Folsomia_quadrioculata_Ita_2_KF684453_3	lineage_36
	Folsomia_quadrioculata_Ita_3_KF684454_3	lineage_36
	Folsomia_quadrioculata_Ita_4_KF684455_3	lineage_36
	Folsomia_quadrioculata_Ita_5_KF684456_3	lineage_36
	Folsomia_quadrioculata_Rus_1_KF684467_3	lineage_31
	Folsomia_quadrioculata_Rus_2_KF684468_3	lineage_31
	Folsomia_quadrioculata_Rus_3_KF684469_3	lineage_31
	Folsomia_quadrioculata_Rus_4_KF684470_3	lineage_31
	Folsomia_quadrioculata_Ser_1_KF684471_3	lineage_38
	Folsomia_quadrioculata_Ser_2_KF684472_3	lineage_38

	Folsomia_quadrioculata_Ser_3_KF684473_3	lineage_38
	Folsomia_quadrioculata_Ser_4_KF684474_3	lineage_38
	Folsomia_quadrioculata_Ser_5_KF684475_3	lineage_38
	Folsomia_quadrioculata_Spa_1_KF684476_3	lineage_41
	Folsomia_quadrioculata_Spa_2_KF684477_3	lineage_41
	Folsomia_quadrioculata_Spa_3_KF684478_3	lineage_41
	Folsomia_quadrioculata_Spa_4_KF684479_3	lineage_41
	Folsomia_quadrioculata_Spa_5_KF684480_3	lineage_41
