# Supplementary figures and images for: Applicability and perspectives for DNA barcoding of soil invertebrates
Source: PeerJ. 2024 Jul 24;12:e17709. doi: 10.7717/peerj.17709 (PMC13043172; doi:10.7717/peerj.17709)

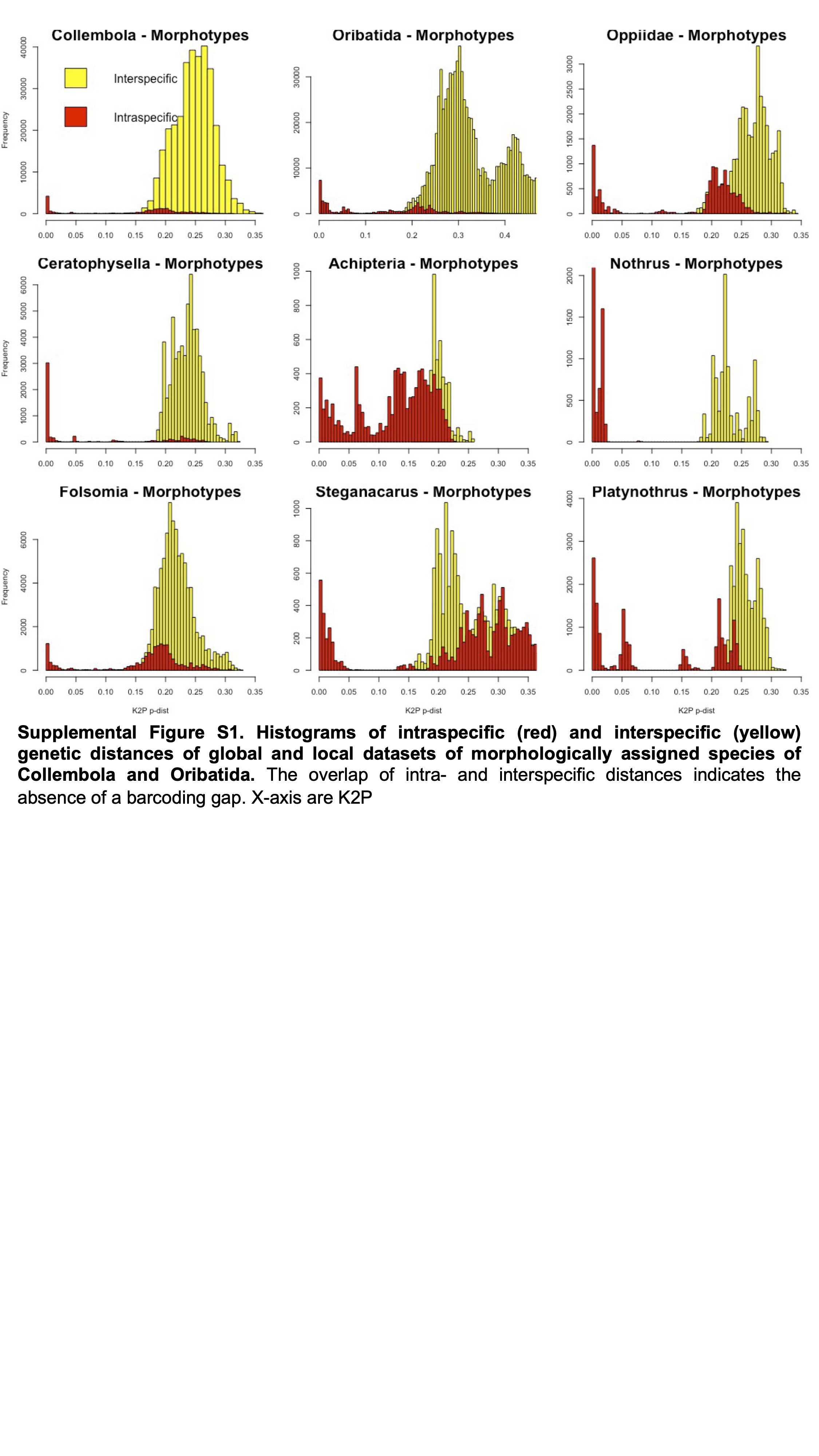

Supplement: Supplemental Information 3 — The overlap of intra- and interspecific distances indicates the absence of a barcoding gap. X-axis are K2P [file peerj-12-17709-s003.jpg]

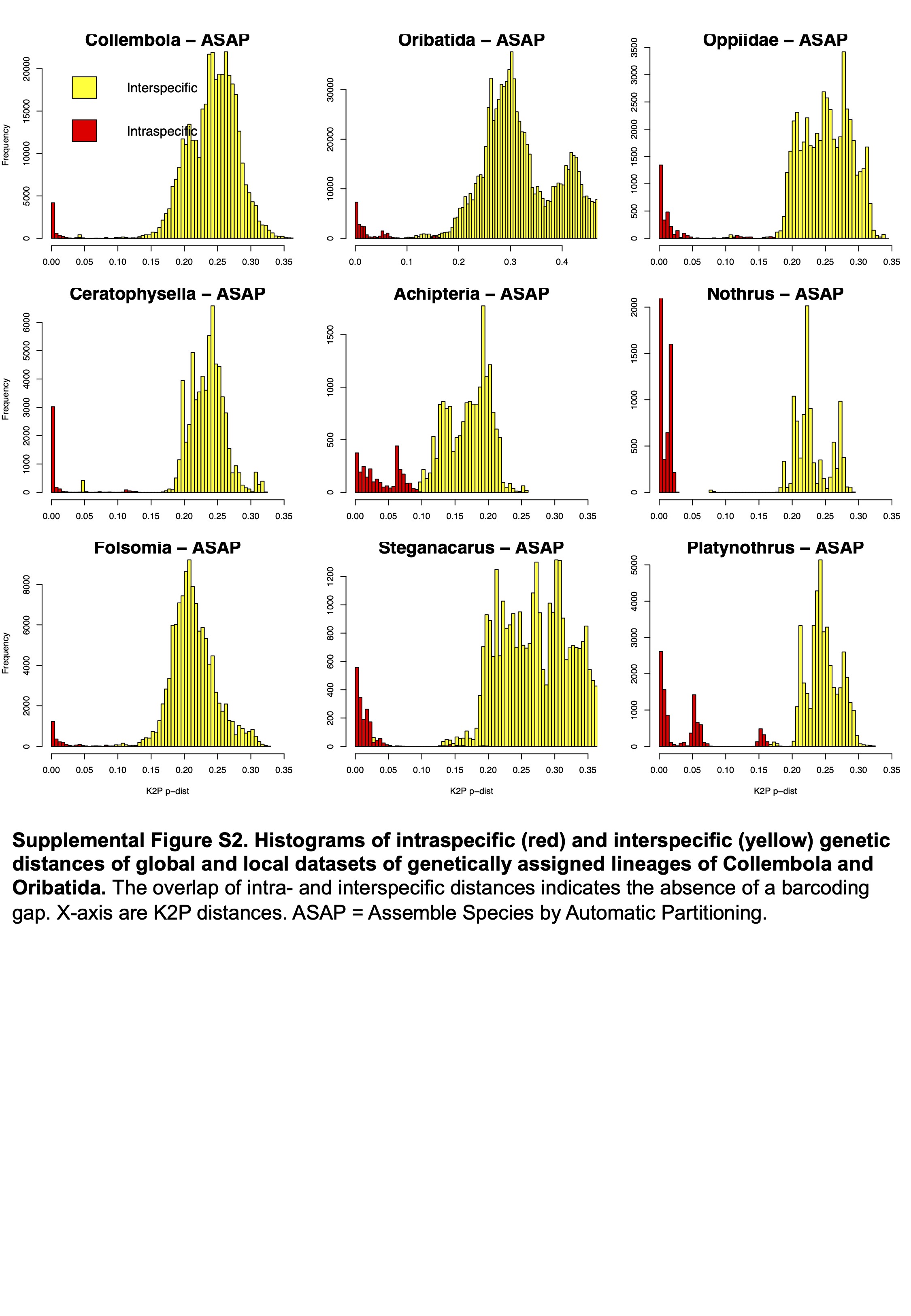

Supplement: Supplemental Information 4 — The overlap of intra- and interspecific distances indicates the absence of a barcoding gap. X-axis are K2P distances. ASAP = Assemble Species by Automatic Partitioning. [file peerj-12-17709-s004.jpg]

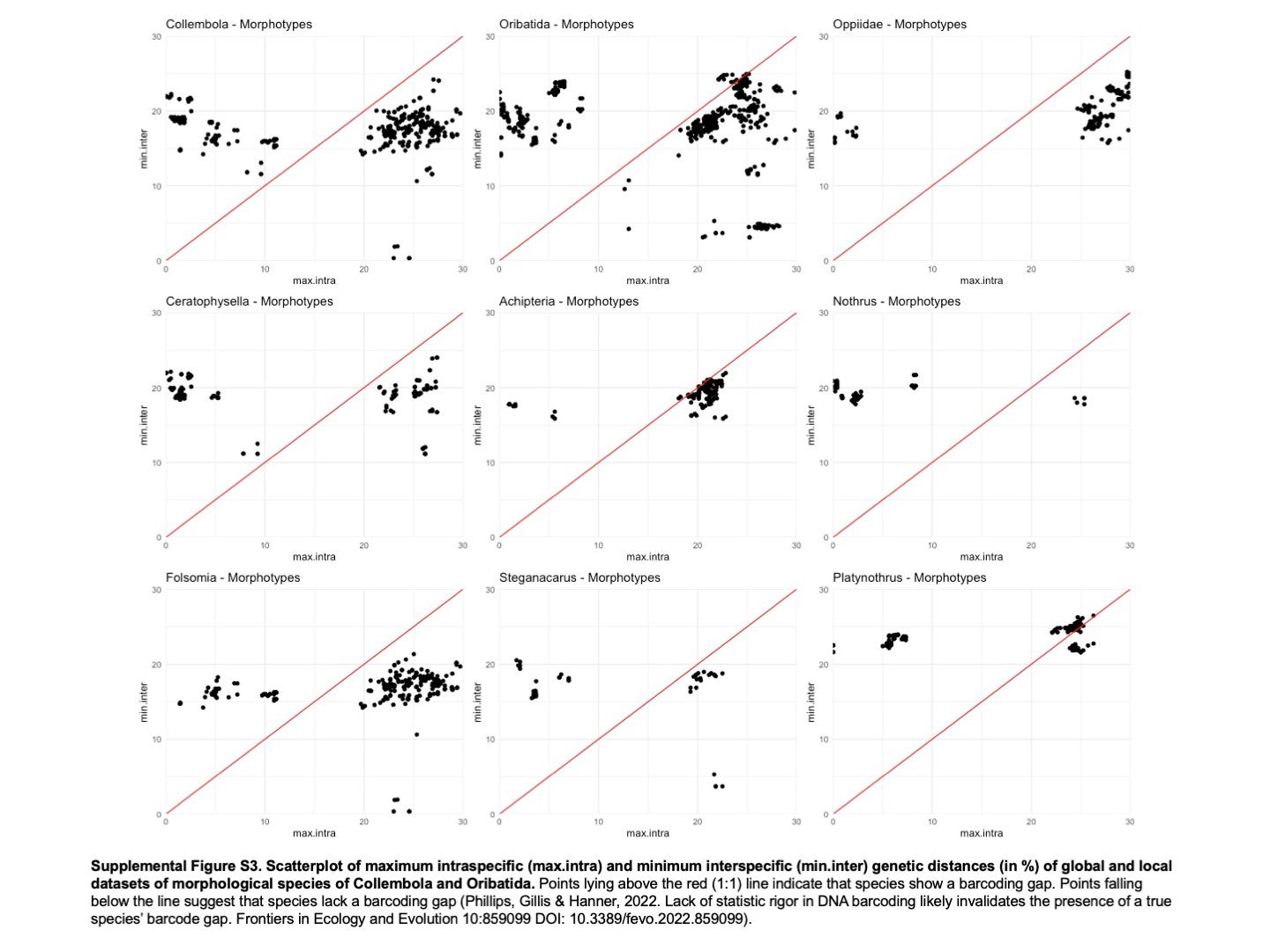

Supplement: Supplemental Information 5 — Points lying above the red (1:1) line indicate that species show a barcoding gap. Points falling below the line suggest that species lack a barcoding gap (Phillips, Gillis & Hanner, 2022). Lack of statistic rigor in DNA barcoding likely invalidates the presence of a true species’ barcode gap. Frontiers in Ecology and Evolution 10:859099 DOl: 10.3389/fevo.2022.859099). [file peerj-12-17709-s005.jpeg]

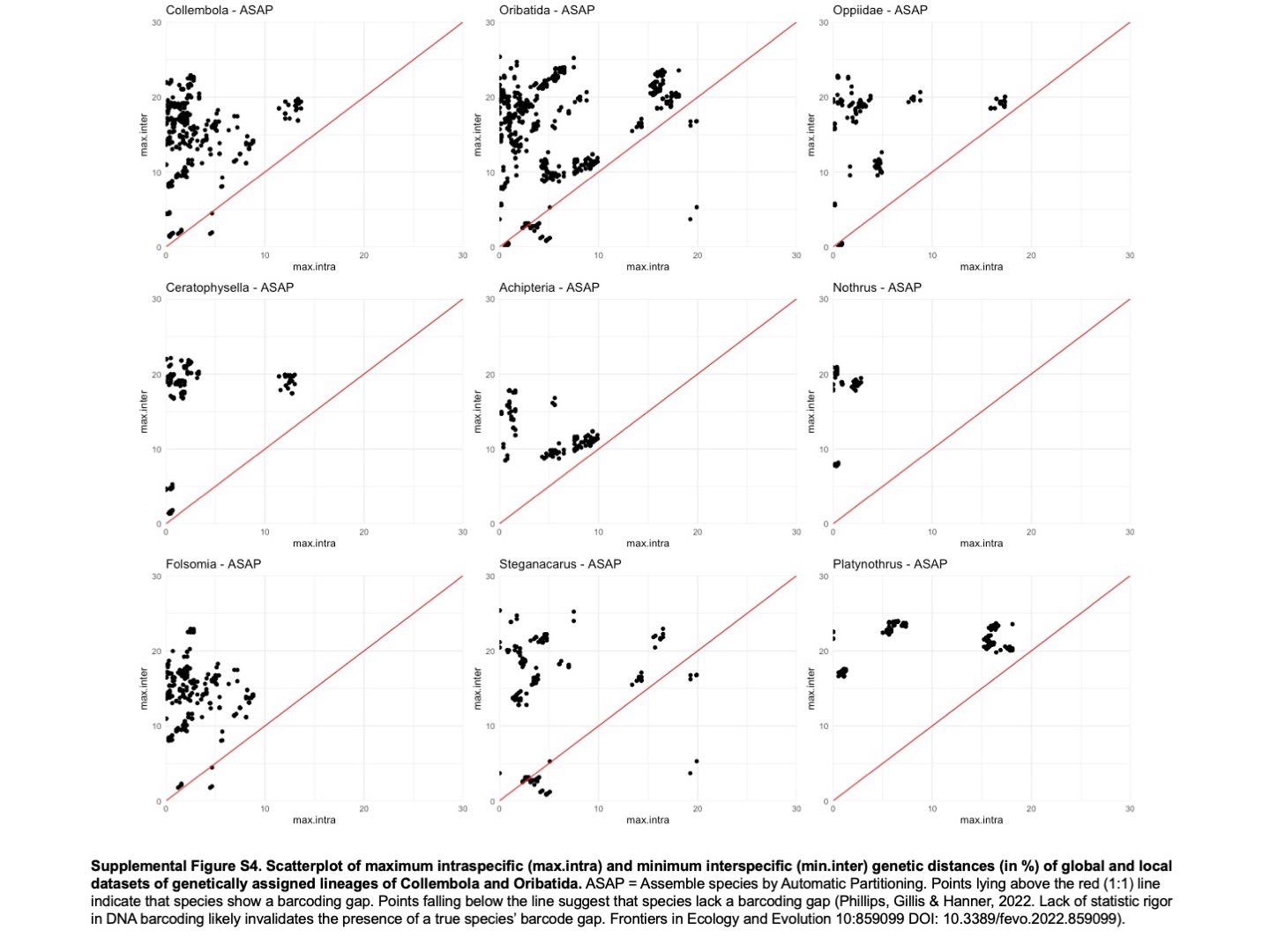

Supplement: Supplemental Information 6 — ASAP = Assemble species by Automatic Partitioning. Points lying above the red (1:1) line indicate that species show a barcoding gap. Points falling below the line suggest that species lack a barcoding gap (Phillips, Gillis & Hanner, 2022). Lack of statistic rigor in DNA barcoding likely invalidates the presence of a true species’ barcode gap. Frontiers in Ecology and Evolution 10:859099 DOl: 10.3389/fevo.2022.859099). [file peerj-12-17709-s006.jpeg]

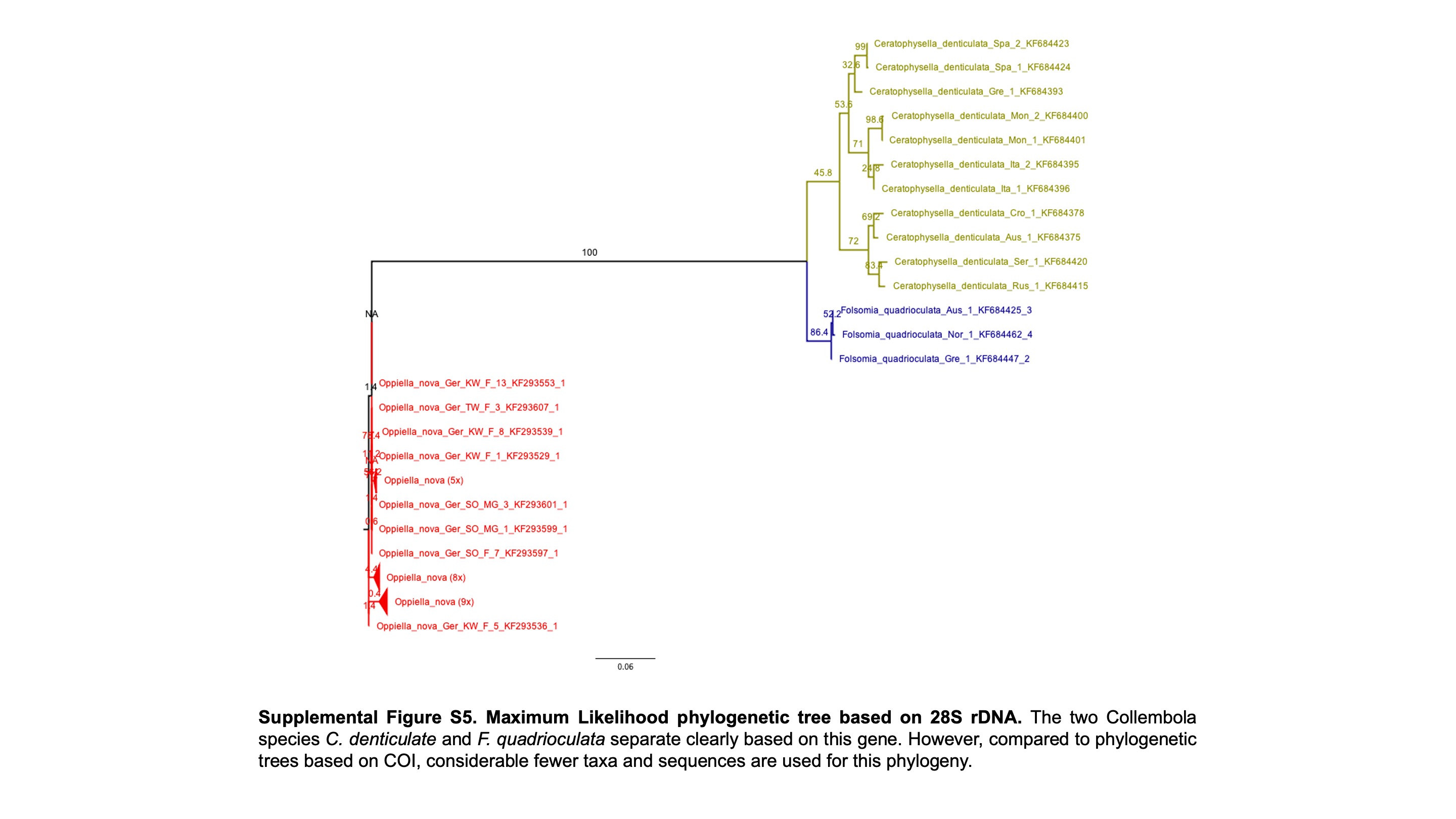

Supplement: Supplemental Information 7 — The two Collembola species C. denticulate and F. quadrioculata separate clearly based on this gene. However, compared to phylogenetic trees based on COl, considerable fewer tax and sequences are used for this phylogeny. [file peerj-12-17709-s007.jpeg]

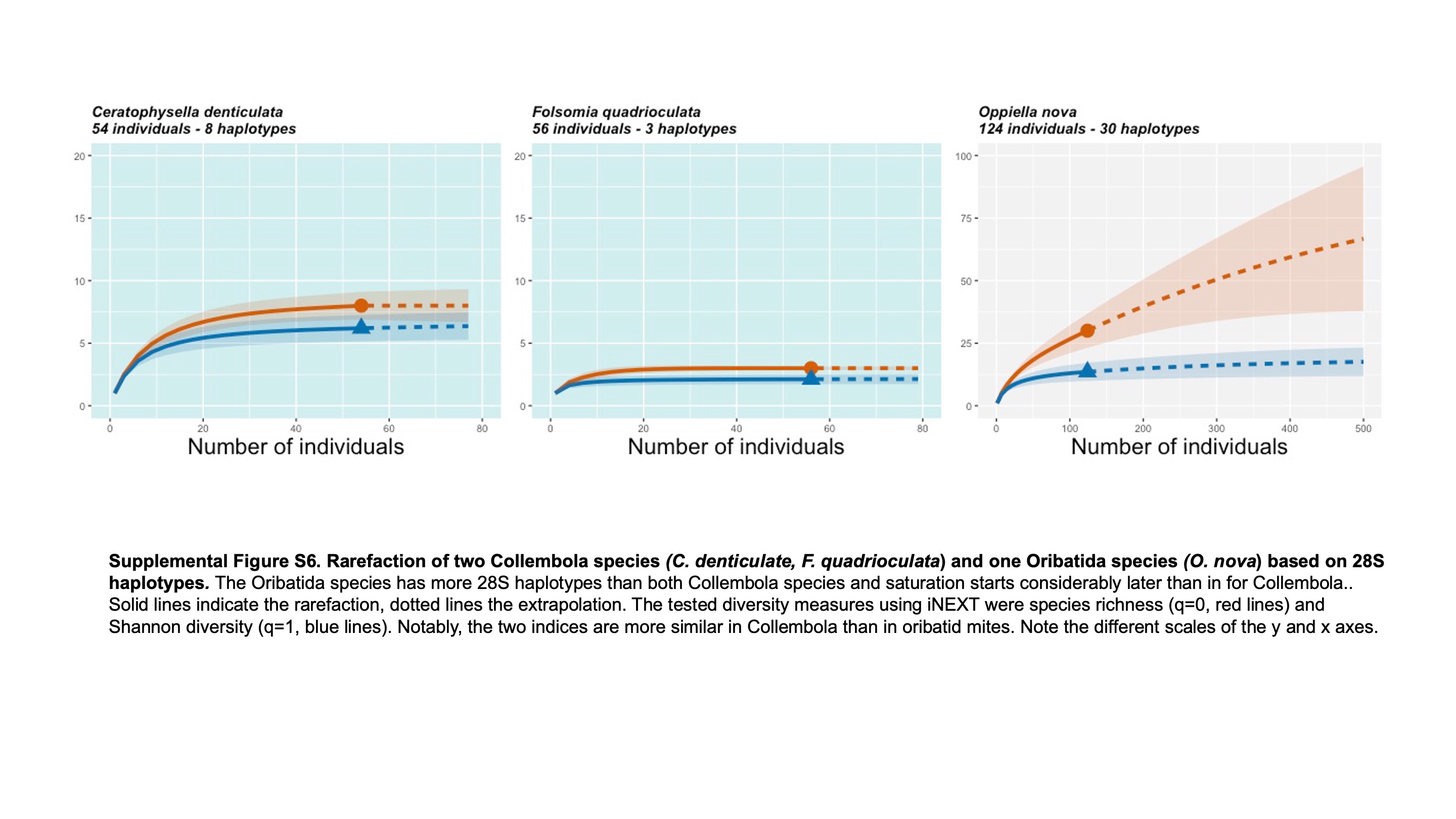

Supplement: Supplemental Information 8 — The Oribatida species has more 28S haplotypes than both Collembola species and saturation starts considerabl later than in for Collembola. Solid lines indicate the rarefaction, dotted lines the extrapolation. The tested diversity measures using iNEXT were species richness (q = 0, red lines) and Shannon diversity (q = 1, blue lines). Notably, the two indices are more similar in Collembola than in oribatid mites. Note the different scales of the y and x axes. [file peerj-12-17709-s008.jpg]
